# Closed-Loop Recycling of 3D-Printed Wood–PLA Composite Parts: Effects on Mechanical and Structural Properties via Fused Filament Fabrication

**DOI:** 10.3390/polym16213002

**Published:** 2024-10-25

**Authors:** Yu-Chen Chien, Jyh-Horng Wu, Chiao-Hsuan Shu, Jung-Tien Lo, Teng-Chun Yang

**Affiliations:** Department of Forestry, National Chung Hsing University, Taichung 402, Taiwan; g111033208@smail.nchu.edu.tw (Y.-C.C.); eric@nchu.edu.tw (J.-H.W.); momoden123@gmail.com (C.-H.S.); lrzhen9163542@gmail.com (J.-T.L.)

**Keywords:** closed-loop recycling, 3D printing, wood fiber, polylactic acid (PLA), fused filament fabrication (FFF)

## Abstract

This study investigated the closed-loop recycling of 3D-printed wood fiber (WF)-filled polylactic acid (PLA) composites via fused filament fabrication (FFF). The WF–PLA composites (WPCs) were extruded into WPC filaments (WPC_f_s) to produce FFF-printed WPC parts (WPC_p_s). The printed WPC_p_s were reprocessed three times via extrusion and 3D-printing processes. The tensile properties and impact strengths of the WPC_f_s and WPC_p_s were determined. To further investigate the impact of closed-loop recycling on the surface morphology, crystallinity, and molecular weight of WPC_f_s, scanning electron microscopy (SEM), differential scanning calorimetry (DSC), and gel permeation chromatography (GPC), respectively, were used. After closed-loop recycling, the surface morphology of the WPC_f_s became smoother, and a decrease in the pore sizes was observed; however, the tensile properties (tensile strength and elongation at break) deteriorated. With increasing numbers of recycling iterations, the molecular weight of the PLA matrix decreased, while an increase in crystallinity was observed due to the recrystallization of the low-molecular-weight PLA molecules after recycling. According to the SEM images of the recycled WPCps, their layer heights were inconsistent, and the layers were rough and discontinuous. Additionally, the color difference (ΔE*) of the recycled WPC_p_s significantly increased. Compared with those of the WPC_p_s after recycling them only once, the tensile strength, elongation at break, and impact strength of the WPC_p_s noticeably decreased after recycling them twice. Considering the changes in various properties of the WPC_f_s and WPC_p_s, the FFF-printed WPC parts can be reprocessed only once through 3D printing.

## 1. Introduction

Additive manufacturing (AM) technologies, known as 3D printing, can fabricate three-dimensional products with complex geometries. According to reports and data [1], the global AM market was 7.97 billion USD in 2018 and is expected to reach 23.33 billion USD by 2026, growing at a high rate of 14.4%. Recently, thermoplastic filament-based material extrusion (ME), commonly called fused deposition modeling (FDM)/fused filament fabrication (FFF), has achieved widespread use owing to its accessibility, versatility, and low cost. However, this printing method can generate substantial waste, such as support materials, disposable prototypes, and failed filaments and parts [2,3]. Recycling is a viable approach to convert waste into new recycled feedstock for further processing. Therefore, the systematic collection and reprocessing of these products and waste play crucial roles. The circular economy for 3D printing serves as an alternative to the traditional linear economy model of “take–make–dispose”, which emphasizes resource reuse, waste reduction, and minimized disposal [2,3,4]. Additionally, various resources are implemented in a closed-loop economic system based on the principles of the six Rs (reduce, reuse, recycle, recover, redesign, and remanufacture) [2,5]. This approach enhances environmental sustainability and reduces production costs, fostering symbiosis between our environment and resources and therefore achieving sustainable development and zero waste. Previous studies have validated the feasibility of utilizing various types of postconsumer recycled plastic waste as feedstocks for ME-type 3D printers [2,4,6,7,8,9]. Therefore, users of ME-type 3D printers can locally recycle plastic waste and reuse it to print new products, bypassing transportation and industrial supply chains. Furthermore, feedstock materials can be reinforced and transformed into useful composites through material extrusion filament and 3D printing methods. The previous studies demonstrated that industrial thermoplastic materials could be recycled and upcycled by incorporating various fibers, optimizing fiber aspect ratios, and controlling fiber orientation, leading to enhanced mechanical properties in the final 3D-printed parts [10,11,12]. Compared with conventional molding methods such as compression molding and injection molding, the distributed recycling of plastic waste through 3D printing is considered one of the novel methods to achieve a circular economy [3,9,13,14].

Polylactic acid (PLA), which is a popular material for 3D printing, has excellent mechanical properties and biodegradability [15]. Furthermore, wood fibers (WFs), as common natural fibers, have been added to PLA as fillers to obtain products or 3D-printed parts with renewability, low density, low cost, and good specific mechanical properties [16,17,18,19,20,21,22]. However, PLA exhibits thermodynamic instability, leading to thermomechanical degradation during multiple processing cycles (e.g., recycling) due to high temperatures and shear forces [23,24,25,26]. Several studies have explored and reviewed the influence of reprocessing on the properties of PLA filaments and their printed part [3,8,13,27]. Romani et al. [8] investigated the thermal, rheological, and mechanical properties of FFF-printed PLA parts after six recycling processes. According to their results, the viscosity, tensile strength, and elongation at the break of PLA significantly decreased due to a decrease in molecular weight after recycling six times; however, the thermal properties slightly changed. Zhao et al. [13] conducted two cycles of recycling for the PLA filaments and the FFF-printed parts. They reported that as the number of recycling cycles increased, the average molecular weight, thermal stability, and viscosity of PLA decreased, whereas the mechanical properties of the printed parts did not significantly change. Gonçalves et al. [27] explored the effects of five cycles of mechanical recycling under drying and nondrying conditions before processing on the acidity, molecular weight, thermal properties, and coloring of PLA. The results indicated that a decrease in molecular weight and an increase in acidity and yellowing were significant with an increasing number of recycling cycles, particularly for PLA, without drying before processing [27]. Additionally, wood is a lignocellulose material that thermally degrades at high temperatures (>150 °C), resulting in changes in chemical composition and physical and mechanical properties [28,29]. Therefore, multiple cycles of processing cause thermal degradation of both the plastic matrix and the wood fibers. To the best of our knowledge, there is little information available on the effects of reprocessing on FFF-printed WF–PLA composite (WPC) parts. Furthermore, heat treatment, which is an environmentally friendly physical treatment, has been applied to WF–plastic composites to improve the dimensional stability and compatibility between WFs and the plastic matrix [20,30]. Accordingly, the aim of the present study was to investigate the reprocessing of PLA composites with heat-treated WFs via FFF. For each recycling process, the thermal stability, molecular weight, surface morphology, density, surface color, tensile properties, and impact strength of the WPC filaments and FFF-printed parts were determined. This research could provide insight into the implementation of distributed recycling for FFF-printed WPC parts, therefore achieving a circular economy in 3D printing.

## 2. Experimental

### 2.1. Materials

Commercial PLA (Ingeo 3D850) pellets were purchased from NatureWorks (Plymouth, MN, USA) and had a specific gravity of 1.24 g/cc, a melt flow rate of 7–9 g/10 min, and a melting temperature of 165–180 °C. Wood fibers (WFs) were obtained from Japanese cedar (*Cryptomeria japonica* D. Don) sapwood in the experimental forest of National Taiwan University (Nan-Tou County, Taiwan), ground, and passed through a 100-mesh sieve. To increase the dimensional stability of the WFs, they were subjected to heat treatment in an oven at 180 °C for 4 h.

### 2.2. Preparation of WPC Filaments and Parts

**Figure 1:**
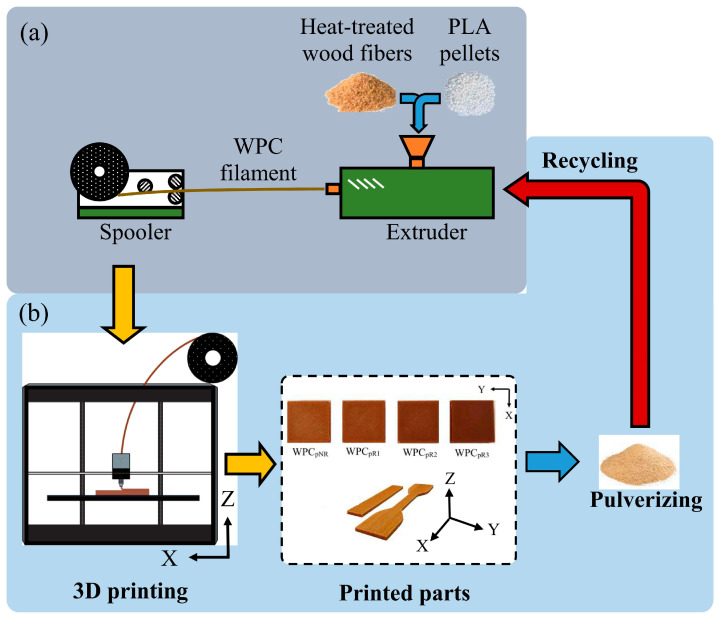
Schematic diagrams of manufacturing for a WPC filament (**a**) and 3D printing and recycling for a WPC part (**b**).

As shown in Figure 1, the WFs and PLA pellets were mixed in a single-screw extruder (EX6 Filament Extruder, Filabot Co., Ltd., Barre, VT, USA) to prepare the WPC mixture.

The temperature was set to 70, 210, 180, and 176 °C from the feed zone to the melting/pumping zone, with a screw speed of 16 rpm, producing WPC filaments (WPC_f_s) with a diameter of 1.65 ± 0.1 mm. Before mixing, the WFs and PLA pellets were dried at 105 °C and 60 °C for 24 h. The ratio of the WFs to PLA was 1:4. All WPC parts (WPC_p_s) were printed via an FFF printer (Creator Pro, Flashforge 3D Technology Co., Ltd., Hangzhou, China) with a nozzle size of 0.6 mm. All the samples were printed parallel to the printing X-axis with a 100% filling pattern, and one layer of printed contour was added around each test sample. The temperatures of the nozzle and heating plate were 210 °C and 60 °C, respectively. Additionally, the layer thickness of the printed WPC_p_s was 0.3 mm, and the printing speed was 30 mm/s. All test samples were conditioned at 20 °C and 65% relative humidity (RH) for one week. After all properties of the WPC_p_s were measured, they were collected, pulverized, and reprocessed under the same manufacturing conditions to obtain recycled WPC_f_s from the extruder. The recycled WPC_p_s were subsequently printed under the same printing conditions, and the process was repeated a total of three times. Based on the number of recycling cycles (nonrecycled (NR) and recycled 1 (R1) to 3 (R3) times), the WPC filaments and parts were named WPCfn and WPCpn, respectively, where n represents the number of recycling cycles (NR, R1, R2, and R3).

### 2.3. Characterization of WPC Filaments and Parts

#### 2.3.1. Differential Scanning Calorimetry (DSC)

The heat flow of recycled WPC filaments (5 mg) was recorded at the glass transition temperature (T_g_), crystalline temperature (T_c_), and melting temperature (T_m_) via a DSC 8500 instrument (PerkinElmer, Beaconsfield, UK). The samples were heated from 20 to 210 °C at 10 °C/min under nitrogen (a flow rate of 20 mL/min). Additionally, the crystallinity index (X_c_) was calculated via the following equation:

(1) $$X_{c}\;(\%)=100\;\times \;\frac{\Delta H_{m}-\Delta H_{\mathrm{cc}}}{\Delta H_{o}\times w_{c}}$$

where ΔH_m_ is the enthalpy of melting crystallization, ΔH_cc_ is the enthalpy of cold crystallization, ΔH_o_ is the enthalpy of melting of 100% crystallized PLA (93 J/g), and w_c_ is the weight fraction of the PLA matrix.

#### 2.3.2. Gel Permeation Chromatography (GPC)

The molecular weight distribution of the PLA matrix in the WPC filament was measured via a GPC with 10E3A/10E4A columns (Phenogel, Phenomenex, Torrance, CA, USA). The various recycled WPC filaments were dissolved in tetrahydrofuran (THF) and filtered through a hydrophilic polypropylene (GHP) membrane to remove wood fibers. The samples were then injected through THF as the isocratic mobile phase at a flow rate of 1 mL/min. Additionally, the number average molecular weight (*M*_n_), weight average molecular weight (*M*_w_), and polydispersity index (PDI) of the WPC filaments after reprocessing were calculated using polystyrene as a calibration standard.

#### 2.3.3. Scanning Electron Microscopy (SEM)

Images of the surface morphologies of the recycled WPC filaments and their failure cross-sectional surfaces after tensile testing were obtained via SEM (TM–1000, Hitachi, Tokyo, Japan) with an accelerating voltage of 15 kV.

#### 2.3.4. Density

In accordance with CNS 13333-1 [31], the density of the printed WPC part (dimensional size: 10 mm (X) × 10 mm (Y) × 5 mm (Z)) was determined via the Archimedes method with a semi-micro analytical balance (GH-200, A&D Co., Ltd., Tokyo, Japan). The samples were immersed in water at 23 °C, and their mass (m_w_) was recorded. The density was calculated via the following equation:

(2) $$\mathrm{Density}\;(g/{\mathrm{cm}}^{3})=\frac{m_{A}\times \delta_{w}}{m_{A}-m_{w}}$$

where m_A_ is the mass of the sample before immersion in water (g), m_w_ is the mass of the sample after immersion in water (g), and δ_w_ is the density of water at 23 °C.

#### 2.3.5. Surface Color

The color parameters of the WPC parts were recorded with a UV–Vis-NIR spectrophotometer (LAMBDA 1050+, PerkinElmer Co., Ltd., Waltham, MA, USA). The color difference (ΔE*) of the recycled WPC part was determined as:

ΔE* = [(L*_1_ − L*_0_)^2^ + (a*_1_ − a*_0_)^2^ + (b*_1_ − b*_0_)^2^]^1/2^ (3)

where L*_1_ and L*_0_ are the white/black values of nonrecycled and recycled WPC_p_s, a*_1_ and a*_0_ are the red/green values of nonrecycled and recycled WPC_p_s, and b*_1_ and b*_0_ are the yellow/blue values of nonrecycled and recycled WPC_p_s, respectively.

#### 2.3.6. Tensile Properties

**Figure 2:**
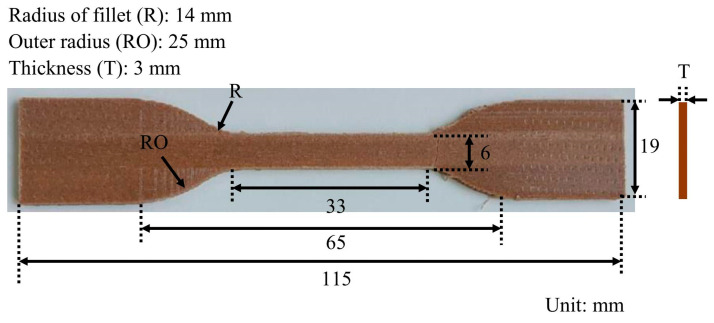
Dimension of the 3D-printed WPC part for tensile test.

The tensile strength (*σ*_f_), tensile modulus (*E*_f_), and elongation at break (*ε*_f_) of the WPC filaments were obtained from tensile tests at a span of 30 mm and a tensile loading speed of 5 mm/min. For the WPC parts, the tensile strength (*σ*_p_), tensile modulus (*E*_p_), and elongation at break (*ε*_p_) were determined according to ASTM D638-14 [32] using printed type IV samples (Figure 2) at a span of 65 mm and a tensile loading speed of 5 mm/min [21].

#### 2.3.7. Impact Strength

In accordance with CNS 5846-1 [33], the Charpy impact strength (IS) of the printed WPC part with unnotched rectangular samples (sample size: 80 mm (X) × 10 mm (Y) × 4 mm (Z)) was evaluated via a YASUDA impact tester (Nishinomiya, Japan). The IS value of the sample was expressed via the equation below:

(4) $$\mathrm{IS}\;(\mathrm{kJ}/m^{2})=\frac{E_{c}}{\mathrm{hb}}\;\times \;10^{3}$$

where E_c_ is the energy absorbed (J), h is the thickness of the sample (mm), and b is the width of the sample (mm).

### 2.4. Analysis of Variance

The significance of the differences among all the recycled samples was calculated via Scheffe’s test (*p* < 0.05).

## 3. Results and Discussion

### 3.1. Properties of the Filaments

**Figure 3:**
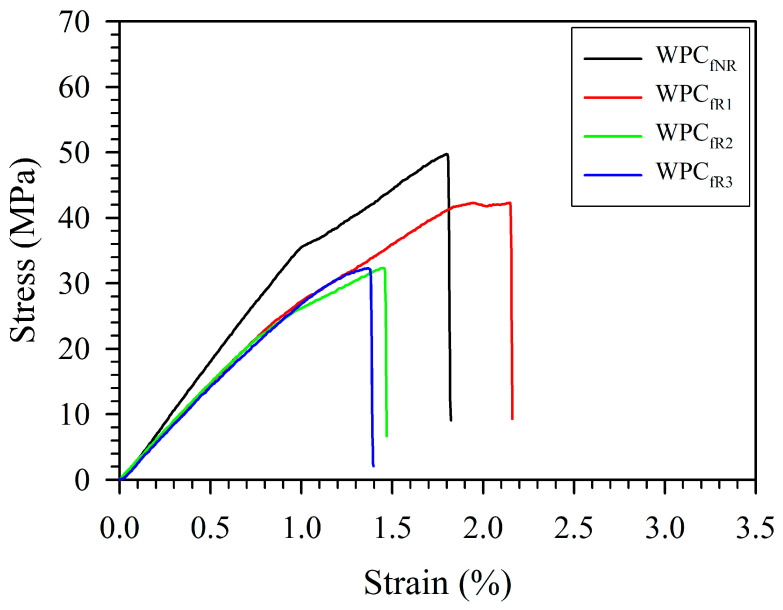
Tensile stress-strain curves of various WPC filaments (WPC_f_s) obtained from the recycling of 3D-printed WPC parts.

The tensile stress-strain curves and tensile properties of various WPC filaments (WPC_f_s) obtained from the recycling of 3D-printed WPC parts (WPC_p_s) are presented in Figure 3 and Table 1.

**Figure 4:**
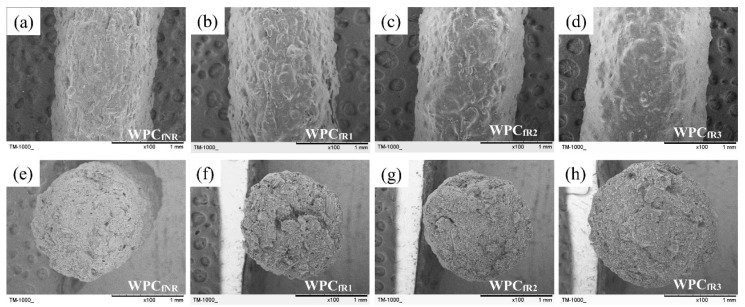
SEM images of surface morphologies (**a**–**d**) and failure cross-sectional surfaces (**e**–**h**) of recycled WPC filaments. (**a**,**e**) WPC_fNR_; (**b**,**f**) WPC_fR1_; (**c**,**g**) WPC_fR2_; (**d**,**h**) WPC_fR3_.

For nonrecycled WPC_f_s (WPC_fNR_), the tensile strength (*σ*_f_), tensile modulus (*E*_f_), and elongation at break (*ε*_f_) were 48.7 MPa, 3.8 GPa, and 2.3%, respectively. After recycling 3 times, the *σ*_f_, *E*_f_, and *ε*_f_ values of the WPC_f_s were reduced to 33.0 MPa, 3.0 GPa, and 1.5%, accounting for 32.2%, 21.1%, and 34.8% reductions, respectively. The results indicated that the tensile properties of the WPC_f_s decreased as the number of recycling cycles increased. According to previous studies, a decrease in the tensile properties of the WPC_fs_ could be attributed to the fact that the characteristics of the PLA matrix and WFs are influenced by multiple processes at high temperatures and mechanical stresses [34,35,36,37,38,39]. Three major steps affect the properties of recycled 3D-printed WPC parts: (1) the extrusion of WPC filaments; (2) the 3D printing of WPC parts; and (3) the pulverizing of 3D-printed WPC parts. During the extrusion of WPC filaments, the degradation of raw materials is caused by heat and shear stress simultaneously. For 3D printing, the WPC filament is reheated to degrade the fiber and the polymeric matrix. Additionally, pulverizing is a process in which materials are subjected to shear stress. It is well known that the polymeric matrix and WFs are susceptible to higher temperatures (above 200 °C) [28,40]. Figure 4 displays the surface morphologies and failure cross-sectional surfaces of WPC filaments obtained from the recycling of WPC parts.

For nonrecycled WPC_f_s (WPC_fNR_s), an uneven surface morphology (Figure 4a) and a few pores in the cross section (Figure 4e) were observed. With increasing numbers of recycling cycles, the surface morphology gradually became smoother, whereas the number of pores on the failure cross-sectional surface significantly decreased. Among all the samples, the WPC_fR3_ exhibited the smoothest surface morphology (Figure 4d) and the fewest pores (Figure 4h). From the cross-sectional surface of the recycled WPC_f_, better fiber coverage by the PLA matrix and fewer pores were observed. This result should have a positive effect on the tensile properties of the WPC filament. However, there is no improvement in its mechanical properties. Therefore, GPC and DSC were further conducted to investigate the crystallinity and molecular weight of the PLA matrix.

**Figure 5:**
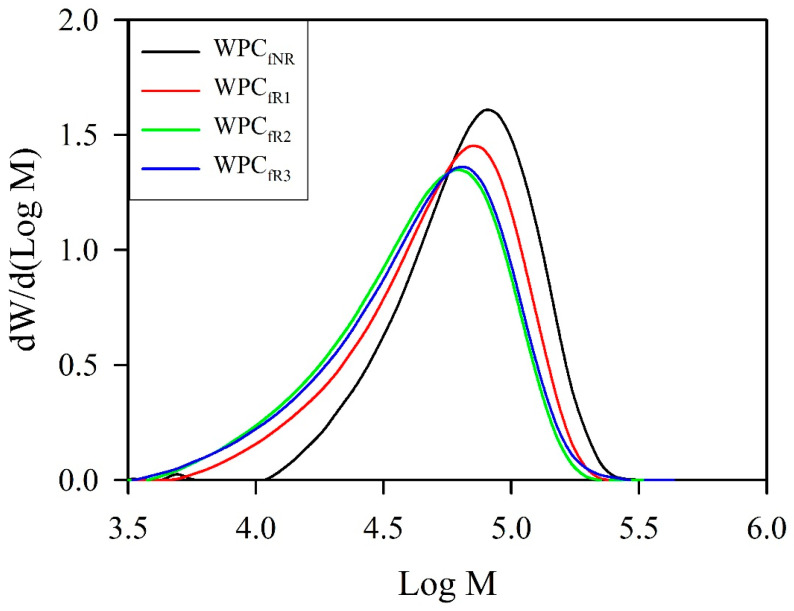
Molecular weight distribution of various WPC filaments obtained from recycling of 3D-printed WPC parts.

Figure 5 and Table 2 present the molecular weight distributions (MWDs) of various recycled WPC filaments and their corresponding values: number average molecular weight (*M*_n_), weight average molecular weight (*M*_w_), and polydispersity index (PDI).

After recycling 2 times, the *M*_n_ and *M*_w_ values of the WPC_f_ significantly decreased from 51.9 kg/mol to 31.5 kg/mol and from 75.0 kg/mol to 52.5 kg/mol, with decreases of 39% and 30%, respectively. However, there were no significant differences in the *M*_n_ and *M*_w_ values after recycling 3 times. The results show that both the *M*_n_ and *M*_w_ values decreased before the third recycling cycle was performed. These reductions in various molecular weights could be related to transesterification and chain scission of the PLA matrix, which occurs via thermomechanical stress during repetitive extrusion of the filaments and 3D-printed parts [8,14,38,39,41,42]. Additionally, the MWD curve gradually shifted to lower molecular weights with an increasing number of cycles (Figure 5). For the distribution of the homogeneity of the PLA chains, the PDI value, which corresponds to *M*_w_/*M*_n_, significantly increased from an initial value of 1.45 to 1.67 after recycling 2 times and then stabilized after recycling 3 times. The PDI value increases because the degree of reduction in the *M*_n_ value is greater than that in the *M*_w_ value owing to the higher probability of chain scission. This mechanism can describe a tendency toward heterogeneity in the length of the PLA chains after recycling. These results are consistent with the literature [8,38,39].

**Figure 6:**
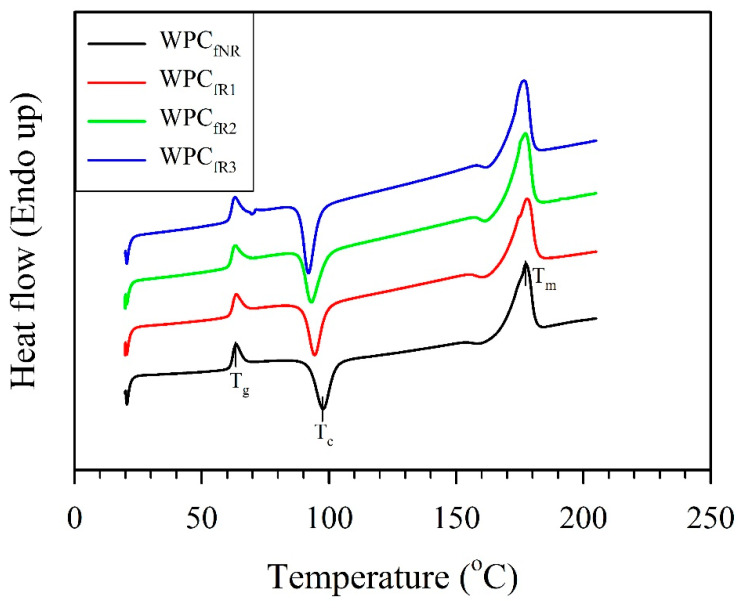
DSC curves of various WPC filaments obtained from recycling of 3D-printed WPC parts.

As illustrated in Figure 6 and Table 3, DSC curves were obtained to investigate the effect of recycling on the thermal properties of the WPC_f_s.

The glass transition temperature (T_g_), crystalline temperature (T_c_), and melting temperature (T_m_) of the nonrecycled WPC_f_ (WPC_fNR_) were 63.4 °C, 97.7 °C, and 177.5 °C, respectively. After recycling 3 times, the T_g_, T_c_, and T_m_ values of the WPC_fR3_ decreased to 62.3 °C, 92.0 °C, and 176.5 °C, respectively. These gradual decreases in all characteristic temperatures can be explained by the better mobility of larger fractions of shortened polymer chains. This phenomenon further decreases the glass transition temperature, cold crystallization temperature, and melting temperature of the recycled WPC_f_s [13,14,43]. In terms of crystallinity (X_c_), the WPC_fNR_ had an X_c_ value of 21.7%. According to a previous study reported by Zhao et al. [13], nonrecycled PLA (PLA_NR_) exhibited a very low X_c_ value (1.17%). Compared with that in the present study, the X_c_ value of PLA with wood fibers was greater than that of PLA_NR_. With the addition of natural fibers, the crystallinity of PLA noticeably increases since fibers act as heterogeneous nucleating agents to promote the crystallization of the PLA matrix [44,45,46]. The X_c_ value of the WPC_f_ gradually increased from 21.7% (WPC_fNR_) to 28.1% (WPC_fR2_) after recycling two times. The results of the GPC analysis (Table 2) revealed that repetitive thermal and mechanical degradation increased the fractions of low-molecular-weight PLA molecules. In addition to the presence of WFs as nucleation sites, the PLA matrix recrystallizes more easily and produces more crystals to increase the X_c_ value of the WPC_f_. An increase in the X_c_ value of recycled PLA has been shown by several studies [13,47]. Interestingly, the X_c_ value of the WPC_f_ decreased to 23.8% after recycling 3 times (WPC_fR3_). As shown by the GPC results (Table 2), there was no further significant reduction in the *M*_w_ and *M*_n_ values of the WPC_fR3_ after recycling 3 times. Therefore, this could be attributed to the greater degradation of natural fiber than to polymeric matrix with repetitive processes [39]. Despite the reduction in the crystallinity of the WPC_fR3_, this value remained higher than that of the nonrecycled WPC_f_ (WPC_fNR_). According to the results of the DSC and GPC analyses, a decrease in the tensile properties of the WPC_f_ may be due primarily to the decrease in the WF strength from thermal degradation and the reduction in the molecular weight of PLA, along with an increase in crystallinity, caused by repeated recycling [39].

### 3.2. Properties of 3D-Printed Parts

#### 3.2.1. Surface Color of the WPC_P_

A colorimetric analysis was performed to measure the color variation to determine the quality of 3D-printed WPC parts (WPC_p_s) after each recycling process. As shown in Table 4, the lightness coordinate (L*) and blue/yellow coordinate (b*) of the WPC_p_s significantly decreased from 49.3 and 25.1 in the nonrecycled WPC_p_ (WPC_pNR_) to 33.0 and 13.4 in the third recycling printed WPC_p_ (WPC_pR3_), respectively.

Additionally, the red/green coordinate (a*) gradually increased from 10.8 in the WPC_pNR_ to 11.5 in the WPC_pR2_, whereas the a* value exhibited the most significant decrease to 9.7 after recycling 3 times. Compared with all the color parameters of the WPC_pNR_, the color difference (ΔE*) significantly increased from 5.5 for the WPC_pR1_ to 20.1 for the WPC_pR3_. This result further indicates that the ΔE* value of the WPC_p_ is influenced mainly by changes in the L* and b* values, suggesting that the surface color of the recycled WPC_p_s tends to become increasingly black and blue. Furthermore, the color changes in the WPC_p_s after recycling multiple times are attributed to the thermal–mechanical degradation of the WFs and the PLA matrix. According to previous studies [8,14,48], the color of PLA shifts toward yellow (an increase in the b* value) and becomes darker (a decrease in the L* value) after each recycling process. Wang et al. [48] and Carrasco et al. [49] reported that thermally degraded PLA has more chromophoric groups (carbon–carbon double bonds conjugated with carbonyls), resulting in a yellowish color. Several studies reported a significant decrease in the L* and b* values of thermally degraded wood, resulting in a darker and bluish color [50,51,52]. The discoloration of thermally degraded wood can be attributed to the formation of chromophoric and auxochromic byproducts such as furfural, hydroxymethyl furfural, dehydrated glucose, quinones, etc. [50,51,52]. As described above, the L* values of both PLA and the WFs tended to decrease after thermal degradation, whereas the b* values exhibited an opposite trend. However, the degree of change in the b* value of the WFs is more significant than that of PLA. Therefore, in the present study, the color of the WPC_p_s after recycling multiple times could be influenced primarily by the color change in the thermally degraded WFs.

#### 3.2.2. Surface Morphology of the WPC_P_

**Figure 7:**
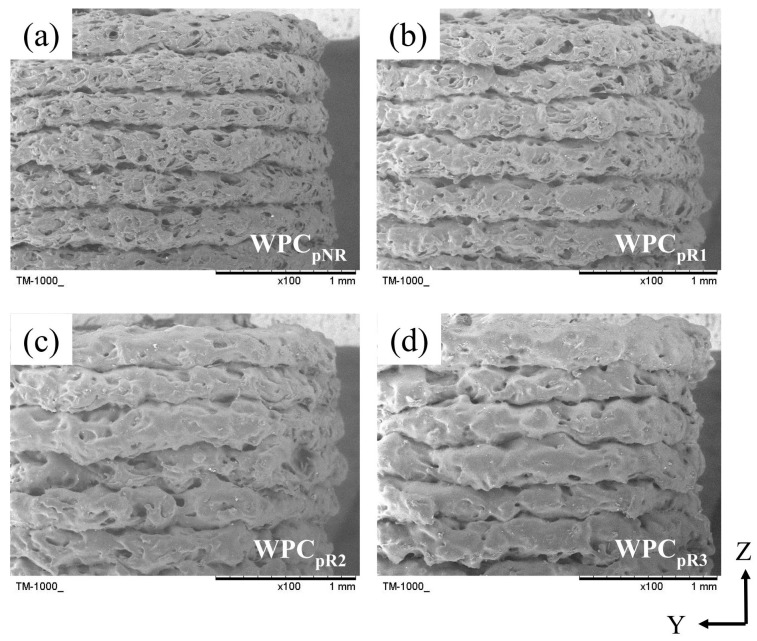
SEM images of layers of 3D printed WPC parts obtained from recycled WPC filaments. (**a**) WPC_pNR_; (**b**) WPC_pR1_; (**c**) WPC_pR2_; (**d**) WPC_pR3_.

Figure 7 compares the layer stacking in the thickness direction (Z axis) between WPC parts obtained after recycling different numbers of times.

Each layer in the WPC_pNR_ exhibited more pores and a uniform layer height (Figure 7a). With the increasing number of recycling processes, there was a significant reduction in the number of pores within the layers. However, the nonuniform layer heights, roughness, and discontinuity of the layers became pronounced (Figure 7b–d). For the recycled WPC_p_, the decrease in the number of pores can be attributed to the greater mobility of the multiprocessed PLA matrix due to their lower molecular weight, as shown by the results of the GPC analysis. Additionally, previous studies imply that uneven melt flows and swelling of the WPC extruded during nozzle extrusion result in a reduced uniformity of printed layer heights and an increase in surface roughness on the layer lines [14,53]. Interestingly, the surface morphology of the recycled WPC_f_ was smoother than that of the layer in the recycled WPC_p_ (Figure 4). This phenomenon may be due to the difference in extrusion dimensions between WPC_f_s (approximately 1.65 mm in diameter) and WPC_p_s (0.6 mm in nozzle diameter), highlighting the influence of WFs and the low viscosity of the PLA matrix. This result indicated that the external surface quality of WPC_p_ is influenced by the number of recycling cycles and the size of the extruded material.

#### 3.2.3. Tensile Properties and Impact Strength of the WPC_P_

The density, tensile properties, and impact strength of the recycled WPC_p_s are listed in Table 5.

The GPC analysis results revealed that the molecular weight of the WPC filaments decreased with an increasing number of recycling iterations (Table 2). This phenomenon was expected to cause an increase in the mass of the WPC parts with increasing recycling time, increasing their density. However, the density of all recycled WPC_p_s ranged from 0.96 to 1.08 g/cm^3^, and no significant difference was observed in density among all samples (Table 5). The results indicated that the density of the WPC_p_s was not significantly influenced by the reduced molecular weight of the WPC_f_s. Liu et al. [14] recycled PLA three times and reported that there was no significant difference in the masses of 3D-printed PLA parts recycled different numbers of times. Additionally, the changes in the tensile properties and impact strength (IS) of the WPC_p_s after reprocessing for three passes are shown in Table 5. Compared with the tensile properties of the nonrecycled WPC_p_ (WPC_pNR_), with an increasing number of recycling cycles, the tensile strength (*σ*_p_) of the WPC_p_s significantly decreased from 31.3 MPa to 24.4 MPa (22.0%), whereas the elongation at break (*ε*_p_) decreased from 1.9% to 1.4% (26.3%). However, there was no significant difference in the tensile modulus (*E*_p_) of the WPC_p_s in the range of 2.8 to 3.4 GPa. Furthermore, a significant decrease of 23.5% in the IS value was observed after recycling 2 times, beyond which the IS value leveled off. Generally, material density has a positive correlation with mechanical properties. However, no significant difference in the density of the recycled WPC_p_s was observed from the results of this study. Therefore, four factors may affect the mechanical properties of recycled and 3D-printed WPC parts [8,13,14,38,39]: (1) degradation of the polymeric matrix; (2) WF degradation; (3) interfacial adhesion between fibers and the matrix; (4) bonding between layers of the extruded materials through the nozzle. As a result of the tensile properties of the WPC_f_s (Table 1), the WPC_pNR_ had the highest *σ*_p_ and *ε*_p_ values among all samples. Regarding the SEM images of the printed samples in Figure 7, the WPC_pNR_ exhibited the highest porosity; however, it still had the highest tensile properties. This result indicated that other factors, such as the molecular weight of the PLA matrix and layer bonding, may play more significant roles in influencing mechanical properties, potentially outweighing the effects of porosity and delamination defects in the nonrecycled WPC_p_. Additionally, a noticeable decrease in the *σ*_p_, *ε*_p_, and IS values of the WPC_p_s could be attributed mainly to the thermomechanical degradation of the WFs and PLA matrix during repetitive extrusion (or 3D printing). These results are similar to those of a previous study reported by Bhattacharjee and Bajwa [39]. This degradation of the PLA and WFs of the recycled WPC_p_s should decrease their *E*_p_ values; however, this phenomenon may be offset by the improved stress transmission due to better bonding between the fibers and the matrix, as well as between the layers of the extruded materials. Therefore, the *E*_p_ values did not significantly differ among all WPC_p_ samples. Without significantly compromising the mechanical properties of the unrecycled WPC parts, the acceptable number of recycling cycles is just one.

## 4. Conclusions

The present study proposed closed-loop recycling via fused filament fabrication based on wood fiber (WF)–PLA composites (WPCs) and investigated extruded WPC filaments (WPC_f_s) and printed WPC parts (WPC_p_s) recycled multiple times through characterization. The results indicated that the tensile strength and elongation at the break of the WPC_f_s significantly decreased with increasing recycling times due to the degradation of the PLA matrix and WFs at high temperatures and with mechanical stress during recycling. The surface morphology of the recycled WPC_f_s gradually became smoother, and a decrease in the number of pores on the failure cross-sectional surface was observed. As the number of cycles increased, the average molecular weight (*M*_w_ and *M*_n_) of the WPC_f_s decreased, resulting in gradual decreases in glass transition temperatures, cold crystallization temperatures, and melting temperatures. Moreover, the crystallinity index of the WPC_f_s increased with an increasing number of recycling times, indicating the recrystallization of the PLA matrix. Compared with those of the nonrecycled WPC_p_s, the lightness coordinate (L*) and blue/yellow coordinate (b*) decreased as the number of recycling times increased, whereas the color difference (ΔE*) significantly increased. From the SEM images of the recycled WPC_p_s, inconsistent heights, roughnesses, and discontinuities were observed on each layer. Additionally, the density and tensile modulus did not significantly differ among all the printed WPC parts. Furthermore, the tensile strength, elongation at break, and impact strength of the printed WPC parts tended to decrease, with significant differences among them with increasing number of recycling times. These findings provide a foundation and reference for research on 3D-printed WPCs and practical applications of the FFF-based multiple recycling method.

## Figures and Tables

**Table 1:** Tensile properties of various WPC filaments obtained from recycling of 3D printed WPC parts.

Code	Recycling Time	*σ*_f_ (MPa)	*E*_f_ (GPa)	*ε*_f_ (%)
WPC_fNR_	-	48.7 ± 6.1 ^a^	3.8 ± 0.2 ^a^	2.3 ± 0.7 ^ab^
WPC_fR1_	1	44.2 ± 1.5 ^ab^	3.0 ± 0.0 ^b^	2.9 ± 0.5 ^a^
WPC_fR2_	2	37.6 ± 4.3 ^bc^	2.9 ± 0.2 ^b^	1.9 ± 0.3 ^bc^
WPC_fR3_	3	33.0 ± 1.2 ^c^	3.0 ± 0.4 ^b^	1.5 ± 0.2 ^c^

Values are the mean ± SD (*n* = 5). Different letters (a, b, and c) within a column indicate significant differences (*p* < 0.05).

**Table 2:** GPC results of various WPC filaments obtained from recylcing of 3D-printed WPC parts.

Code	Recycling Time	*M*_n_ (kg/mol)	*M*_w_ (kg/mol)	PDI
WPC_fNR_	-	51.9 ± 4.1 ^a^	75.0 ± 2.0 ^a^	1.45 ± 0.08 ^c^
WPC_fR1_	1	40.2 ± 1.4 ^b^	62.4 ± 1.0 ^b^	1.55 ± 0.04 ^bc^
WPC_fR2_	2	31.5 ± 1.8 ^c^	52.5 ± 1.3 ^c^	1.67 ± 0.05 ^ab^
WPC_fR3_	3	32.2 ± 1.5 ^c^	55.2 ± 0.8 ^c^	1.72 ± 0.06 ^a^

Values are the mean ± SD (*n* = 4). Different letters (a, b, and c) within a column indicate significant differences (*p* < 0.05).

**Table 3:** DSC results of various WPC filaments obtained from recycling of 3D-printed WPC parts.

Code	Recycling Time	T_g_ (°C)	T_c_ (°C)	T_m_ (°C)	ΔH_cc_ (J/g)	ΔH_m_ (J/g)	X_c_ (%)
WPC_fNR_	-	63.4	97.7	177.5	26.7	42.8	21.7
WPC_fR1_	1	63.5	94.4	177.8	25.3	45.3	26.9
WPC_fR2_	2	63.1	93.1	177.2	26.5	47.4	28.1
WPC_fR3_	3	62.3	92.0	176.5	27.0	44.8	23.8

**Table 4:** Color parameters of various 3D-printed WPC parts obtained from recycled WPC filaments.

Code	Recycling Time	L*	a*	b*	ΔE*
WPC_pNR_	-	49.3 ± 0.6 ^a^	10.8 ± 0.6 ^b^	25.1 ± 0.3 ^a^	-
WPC_pR1_	1	44.2 ± 0.1 ^b^	11.4 ± 0.1 ^a,b^	23.4 ± 0.3 ^a^	5.5 ± 0.2 ^c^
WPC_pR2_	2	38.7 ± 1.2 ^c^	11.5 ± 0.1 ^a^	19.3 ± 1.3 ^b^	12.1 ± 1.7 ^b^
WPC_pR3_	3	33.0 ± 0.3 ^d^	9.7 ± 0.3 ^c^	13.4 ± 0.7 ^c^	20.1 ± 0.7 ^a^

Values are the mean ± SD (*n* = 3). Different letters (a, b, c, and d) within a column indicate significant differences (*p* < 0.05).

**Table 5:** Density, tensile properties, and impact strength of various 3D-printed WPC parts obtained from recycled WPC filaments.

Code	Recycling Time	Density (g/cm^3^)	Tensile Properties			IS (kJ/m^2^)
			*σ*_p_ (MPa)	*E*_p_ (GPa)	*ε*_p_ (%)	
WPC_pNR_	-	1.03 ± 0.03 ^a^	31.3 ± 1.5 ^a^	2.8 ± 0.4 ^a^	1.9 ± 0.0 ^a^	6.8 ± 0.0 ^a^
WPC_pR1_	1	0.96 ± 0.07 ^a^	31.7 ± 1.3 ^a^	3.1 ± 0.4 ^a^	1.9 ± 0.0 ^ab^	6.7 ± 0.9 ^a^
WPC_pR2_	2	1.04 ± 0.04 ^a^	26.4 ± 1.4 ^ab^	3.0 ± 0.1 ^a^	1.7 ± 0.1 ^b^	5.2 ± 0.2 ^b^
WPC_pR3_	3	1.08 ± 0.04 ^a^	24.4 ± 3.7 ^b^	3.4 ± 0.3 ^a^	1.4 ± 0.1 ^c^	5.5 ± 0.2 ^b^

Values are the mean ± SD (*n* = 3). Different letters (a, b, and c) within a column indicate significant differences (*p* < 0.05).

## Data Availability

Data is available on request from the authors.
